# Same Game, Different Names: Cream-Skimming in the Post-ACA Individual Health Insurance Market

**DOI:** 10.1177/0046958020933765

**Published:** 2020-07-09

**Authors:** Daniel W. Sacks, Coleman Drake, Jean M. Abraham, Kosali Simon

**Affiliations:** 1 Indiana University Bloomington, USA; 2 University of Pittsburgh, PA, USA; 3 University of Minnesota, Minneapolis, USA

**Keywords:** Patient Protection and Affordable Care Act, health insurance, individual health insurance market, state insurance policy, cream-skimming, health insurance marketplaces

## Abstract

One of the Affordable Care Act’s (ACA) signature reforms was creating centralized Health Insurance Marketplaces to offer comprehensive coverage in the form of comprehensive insurance complying with the ACA’s coverage standards. Yet, even after the ACA’s implementation, millions of people were covered through noncompliant plans, primarily in the form of continued enrollment in “grandmothered” and “grandfathered” plans that predated ACA’s full implementation and were allowed under federal and state regulations. Newly proposed and enacted federal legislation may grow the noncompliant segment in future years, and the employment losses of 2020 may grow reliance on individual market coverage further. These factors make it important to understand how the noncompliant segment affects the compliant segment, including the Marketplaces. We show, first, that the noncompliant segment of the individual insurance market substantially outperformed the compliant segment, charging lower premiums but with vastly lower costs, suggesting that insurers have a strong incentive to enter the noncompliant segment. We show, next, that state’s decisions to allow grandmothered plans is associated with stronger financial performance of the noncompliant market, but weaker performance of the compliant segment, as noncompliant plans attract lower-cost enrollees. This finding indicates important linkages between the noncompliant and compliant segments and highlights the role state policy can play in the individual insurance market. Taken together, our results point to substantial cream-skimming, with noncompliant plans enrolling the healthiest enrollees, resulting in higher average claims cost in the compliant segment.

**What do we already know about this topic?**

Adverse selection is a concern in the individual insurance market, and premiums have risen rapidly in recent years.

**How does your research contribute to the field?**

We show that state policy decisions—and in particular their decision to allow continued enrollment in Affordable Care Act noncompliant health insurance plans in the individual insurance market—are highly associated with health care costs of Affordable Care Act-compliant plans.

**What are your research’s implications toward theory, practice, or policy?**

Current policy to permit the sale of noncompliant plans may have an impact on premiums of compliant plans.

## Introduction

The Health Insurance Marketplaces are one of the signature features of the Affordable Care Act (ACA). They represent centralized platforms for individuals to purchase standardized insurance plans meeting minimum coverage standards. Such plans, known as “qualified health plans,” (QHPs) cannot be medically underwritten, meaning preexisting conditions cannot be a basis for coverage denials or premium increases. Yet, millions of Americans continue to hold individual insurance market plans outside of the Marketplaces, obtained through various channels, including directly from insurers with or without the assistance of brokers as well as through online portals like ehealthinsurance.com. Especially important are 2 types of off-Marketplace plans: “grandfathered” and “grandmothered” plans. Grandfathered plans are ones offered prior to the ACA’s initial implementation in 2010, and federal regulations permit continued enrollment in them. Grandmothered plans are ones offered between 2010 and the ACA’s full implementation in 2014, and states determine whether these plans can remain in effect. Grandfathered and grandmothered plans are by definition not compliant with the ACA’s coverage standards, they are ineligible for premium subsidies, and they did not count as creditable coverage for the individual mandate, when it existed. But they are also relatively low premium and may therefore be attractive to some enrollees.

Off-Marketplace coverage, especially in the noncompliant segment, is set to expand in future years, as recent regulatory and legislative actions at the federal level have encouraged growth of non-ACA-compliant plans. Through an executive order issued in October 2017, the Trump Administration extended the maximum duration of short-term, limited-duration plans from 3 to 12 months and allowed plans to be renewed for up to 36 months. In that same executive order, the Administration also sought an expanded role for association health plans, which allow small employers, the self-employed, and individuals to purchase plans offered by “associations” of business or trade associations.^1^ As a further push toward noncompliant plans, the Tax Cuts and Jobs Act of 2017 effectively eliminated the individual mandate requirement that most individuals have a compliant plan, thus reducing any penalty for holding non-ACA-compliant coverage. Although noncompliant plans may be appealing to the currently uninsured, the growth of this segment may be of concern, especially if it comes at the expense of the enrollees purchasing compliant coverage, as noncompliant plans often offer inferior financial protection. There is also likely an overall growth that would occur in marketplace coverage if there are extended job losses and losses of employer-sponsored health insurance that occur as a result of COVID-19’s impact on the economy.

We examine the financial performance of compliant and noncompliant individual market plans, and study how state policies affect both segments. Our first finding is that noncompliant plans substantially outperform compliant plans. Noncompliant plans, on average, post lower premiums, have lower claims, and receive fewer subsidies (because they are ineligible for the ACA’s main subsidies). This finding on relative performance fills a gap in prior research on financial performance in the post-ACA individual market, which has either pooled compliant and noncompliant plans in the off-Marketplace segment,^2,3^ or only examined compliant off-Marketplace plans.^4^

Our second finding is that the gap between compliant and noncompliant plans is especially sensitive to a state policy decision: whether or not grandmothered plans can continue to operate. Other state policies—Medicaid expansion and the use of state-based Marketplaces—are not significantly associated with the performance of compliant or noncompliant plans. To our knowledge, the only other evidence of how state policy influences the performance of the individual market either uses data only through the first half of 2014 and pools the compliant and noncompliant portions, or only looks at the compliant portion in 2014 and 2015; this research also finds that state policy decisions meaningfully affected the individual market.^5,6^

These findings imply that the impressive performance of noncompliant plans reflects, in part, their success in cream-skimming, and these plans therefore exert a negative spillover on the compliant plans and their enrollees. This is because noncompliant plans are mostly pre-ACA grandmothered and grandfathered plans, which generally have low premiums and low benefit generosity. Such plans have been able to retain very low-cost enrollees, potentially drawing them away from joining the compliant segment, especially the Marketplaces. Regulations to expand the noncompliant segment may therefore result in higher costs and premiums in the Marketplaces relative to non-Marketplace plans, as we find that grandmothering regulations raised the premiums of compliant plans, relative to noncompliant plans. Although most Marketplace enrollees are subsidized and largely insulated from premium increases,^7^ higher Marketplace premiums have 2 important consequences. First, they decrease the affordability of ACA-compliant health coverage for enrollees who do not qualify for premium tax credit subsidies. Second, they impose a greater financial burden for taxpayers in the form of higher premium tax credit subsidies, which increase dollar-for-dollar with premium increases.

## Methods

### Data Sources

We used 2012-2017 data from insurers’ regulatory filings that document whether they met minimum medical loss ratio (MLR) requirements.^8^ These are the most recent comprehensive individual market data available to date, and they record member months of enrollment, premium revenue, and medical claims costs, as well as payments to and subsidies from several ACA-related programs, including risk corridors, reinsurance, risk adjustment, Marketplace fees, and advanced cost-sharing reduction payments which subsidize low cost-sharing for low-income enrollees. There are separate reports by state, insurer, and market (ie, individual market, small group, large group, and mini-medical coverage). The data contain records of 9696 insurer-state-years with individual market enrollment. These data have been used in other research on the ACA.^4,9,10^

We also used 3 supplemental data sources. First, we obtained information on states’ policies toward grandmothered plans from healthinsurance.org.^11^ We provide more information about the state grandmothering data in the Supplemental Material, where we also provide evidence on their validity. Second, we used data on states’ decisions to expand Medicaid and operate a state-based Marketplace from the Kaiser Family Foundation.^12,13^ Last, we identified Medicaid managed care insurers as those insurers that offer Medicaid managed care plans, according to listings from the Kaiser Family Foundation’s^14^ Medicaid Managed Care Market Tracker.

## Measures

Individual market plans include those sold in the Marketplaces and those sold off-Marketplace. Marketplace plans are necessarily QHPs, meaning that they meet the minimum coverage standards set out in the ACA, and are qualified to be offered in the Marketplaces. QHPs can also be sold off-Marketplace, but off-Marketplace plans can include non-QHP plans as well. We refer to QHPs as “ACA-compliant” plans, and other plans as “noncompliant.” (Note that our definition of noncompliant includes all plans whose benefit designs do not comply with ACA regulations. Some of these plans counted as coverage for the purposes of the now-defunct individual mandate, and some sources would therefore define these plans as compliant.) Noncompliant plans continue to retain their pre-ACA enrollees because of grandfathering and grandmothering regulations, but these plans cannot take new enrollees. A small amount of noncompliant enrollment is in short-term plans, which can take new enrollees. Our data exclude short-term plans.

The MLR data allow us to distinguish between ACA-compliant and noncompliant plans. Specifically, the MLR data contain fields used for calculating risk corridors payments. The risk corridors program was 1 of 2 transitional programs (in addition to reinsurance) created by the ACA to help the Marketplaces reach equilibrium in their initial years of implementation. It was a profit-sharing program between insurers and the government, with transfers to insurers with high claims relative to premiums, and from insurers with low claims relative to premiums.^15^ Only ACA-compliant plans are eligible for these payments, so we observe enrollment, claims costs, and premiums for ACA-compliant plans (aggregated to the insurer-state level). We obtain noncompliant plan data as the difference between individual market totals and the compliant plan totals. The risk corridors program only operated from 2014-2016, so we can distinguish between compliant and noncompliant plans only in these years. We dropped the handful of insurers with negative noncompliant plan enrollment.

We used these plan data to construct insurer-year-segment-level aggregates. Our key measures are total member-years, premium revenue per member month, claims costs per member month, and the markup per member month. For example, we measure premium revenue per member month for Anthem in Indiana in 2014, separately in the compliant and noncompliant segment. We defined markups as premiums earned plus advanced cost-sharing reduction subsidies, as well as risk adjustment transfers, and reinsurance payments received, less medical claims costs paid, per member month. This measure captures the major revenue sources and medical costs of these plans. However, it has 2 limitations. First, we observe advanced cost-sharing reduction subsidies, but not the final payments or reconciliation payments. Second, we do not observe nonclaims costs such as utilization management or marketing expenses, although these are likely very small relative to claims costs, perhaps 1%.^16^ Finally, we imputed segment-specific MLRs, defined as the claims costs less subsidies, divided by premiums less taxes. As we do not observe segment-specific taxes, we prorate the taxes according to each segments share of enrollment.

### Statistical Analysis

To investigate the association between states’ decisions to allow grandmothered plans and each of our 4 key measures, we estimated insurer-state-year-level multivariate linear regressions. We estimated separate regressions for compliant and noncompliant plans. Each outcome measure was regressed on state policy decisions, insurer type indicators, and year and state fixed effects. State policy decisions included states’ decisions to allow grandmothered plans, to expand Medicaid, and to operate a state-based Marketplace. Insurer type indicators identified whether insurers were Blue Cross Blue Shield affiliates, big four insurers (Aetna, Cigna, Humana, and UnitedHealth Group), and Medicaid managed care (ie, an indicator for whether the insurer was a Medicaid managed care insurer). We weighted insurer-year-segment observations by their member-years, and clustered standard errors by state. We limit the sample to state-insurer-segments with at least 1000 member-years, consistent with prior research.^17^

## Results

**Figure 1.**
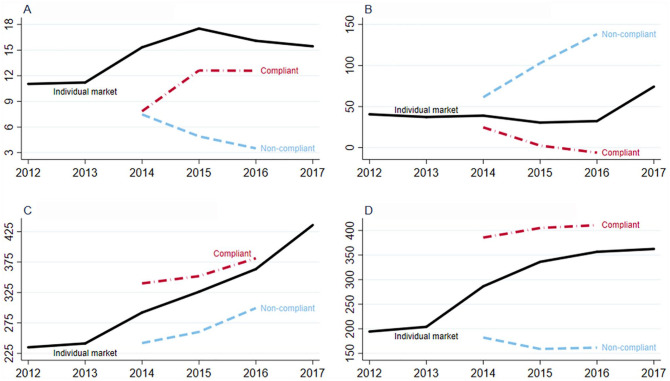
Individual market performance: Markups, members, premiums, and claims. (A) Member-years (millions). (B) Markups per member month ($). (C) Premiums per member month ($). (D) Claims costs per member month ($). *Note.* Markups are defined as premiums earned, ACA-related reinsurance payments received, and cost-sharing reductions subsidies received, less medical claims costs paid, net risk adjustment payments, and Exchange fees paid, per member month. Premiums earned include premiums paid by enrollees and advance premium tax credit payments from the Federal government. Compliant and noncompliant statistics are only reported for 2014-2016 because those are the years in which Risk Corridor payment data were available. ACA = Affordable Care Act.

Figure 1 reports member-years of enrollment, markups per member month, premiums per member month, and claims costs per member month from 2012 to 2017. These measures are reported separately for the compliant and noncompliant segments from 2014 to 2016. The individual market grew substantially between 2012 and 2015, from 11.2 million covered lives to 17.6 million. It then declined through 2017, falling to 15.5 million covered lives. Enrollment growth in 2015 was concentrated in compliant plans, where enrollment grew from 7.8 million covered lives to 12.6 million in 2015. Noncompliant enrollment declined in 2015 and 2016. As noncompliant enrollment is in grandfathered and grandmothered plans, much of this decline likely represents the gradual departure of and exit from such plans. Even in 2016, however, noncompliant-segment enrollment remained substantial, representing approximately 20% of the overall individual market. This large enrollment has the potential to affect costs, premiums, and financial performance in the compliant segment, if the noncompliant-segment enrollees are sufficiently healthier.

On average, across both segments, markups dipped slightly in 2015 and 2016 before improving beyond pre-ACA levels in 2017. However, this across-segment average masks substantial differences between the 2 segments. Compliant plans performed poorly in 2014, with markups of about $25 per member month. Insurers barely covered their medical expenses in 2015 before turning negative in 2016. Noncompliant plans did vastly better. In 2014, average markups were about $60 per member month, and they more than doubled by 2016 to $137 per member per month. The large difference in markups is reflected in a much lower (imputed) MLRs for noncompliant plans than for compliant plans, about 65% versus 90%. We plot these ratios Supplemental Figure 1. We caution, however, that these loss ratios are imputed and may not exactly correspond to the statutory loss ratios.

Premiums per member month grew rapidly, nearly doubling between 2012 and 2017. The fastest periods of premium growth for the overall individual market were in 2014 and 2017, with about 18% growth; in 2015 and 2016, premiums grew by about 11% annually. Average premiums were much higher in the compliant segment than in the noncompliant segment. Premiums among compliant plans grew, on average, by about 7% annually, and slower than overall premium growth. These growth rates were lower than what was often reported in the mainstream media,^18^ because as premiums rose, enrollees substituted toward less expensive plans.^19^

Medical claims costs rose with premiums, rising rapidly in 2014, but not in 2017. Compliant plans have vastly higher claims costs than noncompliant plans. Whereas compliant plans reported costs of about $400 per member per month, noncompliant plans had costs of $175 per month or lower. Thus, the high markups evident in Figure 1 were driven by the very low claims costs of noncompliant plans, rather than high premiums.

It is not necessarily surprising that premiums earned were so different between the 2 segments, even though ACA regulations require that insurers use a common risk pool in setting premiums in a given market.^20^ The noncompliant segment consisted of plans whose premiums are not governed by ACA regulations, and instead were potentially medically underwritten.^21^ They also likely had dramatically different benefit designs. Grandmothered plans remained active in 36 states as of 2016.^11^

State grandmothering decisions turn out to account for a great deal of the difference between the compliant and noncompliant segments, as Table 1 shows. The table reports average markups, premiums, and claims costs in 2016, separately by state grandmothering decisions and by segment (averaging across states and plans). This table shows that the difference in average markups and average claims costs between the 2 segments was much smaller in states without grandmothering than in states with grandmothering. Indeed, the difference in markups in nongrandmothering states was less than half the difference in grandmothering states. Importantly, state grandmothering decisions are associated with higher costs for compliant plans, showing that state policy decisions toward the noncompliant segment potentially affected the compliant segment.

**Table 1.** Insurers’ Financial Performance by Grandmothering Decisions and Market Segment, 2014-2016.

	Compliant	Noncompliant	Difference
A. Markup per member month ($)			
With grandmothering	6.3	150.4	144.2***
Without grandmothering	–4.5	59.6	64.1*
		Difference	80.1**
B. Premiums per member month ($)			
With grandmothering	382.3	284.3	–98.1***
Without grandmothering	379.0	340.6	–38.3
		Difference	–59.7
C. Claims costs per member month ($)			
With grandmothering	421.4	134.6	−286.7***
Without grandmothering	390.2	284.2	−106.0***
		Difference	−180.7**
D. ACA-related transfers per member month ($)			
With grandmothering	45.3	0.8	−44.5***
Without grandmothering	6.7	3.1	−3.5
		Difference	−40.1

*Note.* Table reports the average markup per member month, premiums per member month, and claims costs per member, in states with and without grandmothering regulations in effect, in 2016, separately for compliant and noncompliant individual insurance market plans. Sample consists of 1226 insurers. Grandmothered plans are non-ACA-compliant plans that were created prior to the implementation of the ACA in 2014. Averages are weighted by enrollment. For inference, we estimate regressions of the indicated outcome (markup per month, etc), on dummy variables for “with grandmothering,” “noncompliant,” and their interaction. We estimate robust standard errors, cluster at the state level. ACA = Affordable Care Act.

Statistical significance is indicated by *P < .05. **P < .01. ***P < .001.

The comparison in Table 1 is useful for understanding the effect of state grandmothering decisions because it compares different segments in the same state and year, implicitly holding fixed many factors that might be expected to affect markups in the individual insurance market, such as provider payment rates, typical health care utilization patterns, or Medicaid expansion decisions.^22^ However, the analysis does not account for policies that might have a differential effect on the compliant market, relative to the noncompliant market, nor does it let us measure the effects of policies other than grandmothering.

We therefore expand the analysis and estimate multivariate models in Table 2. This analysis simultaneously investigates the association between segment financial performance and 3 state policy levers: grandmothering, Medicaid eligibility expansions, and use of a state-based marketplace. We let the effect of these policies be different for compliant and noncompliant plans, and we include state fixed effects and year dummies to account for cross-state heterogeneity and common trends. These state fixed effects implicitly control for time-invariant state policy decisions, including for example whether states opted for a modification of the standard age curve to determine premiums. We also control for a limited set of insurer characteristics. In this analysis, the effect of grandmothering is identified by the 3 states that changed their grandmothering policies during the 2014-2016 period: New Mexico (eliminated for 2015 and beyond) and Colorado and Oregon (eliminated for 2016 and beyond).

**Table 2.** Associations Between State Policy Decisions and Individual Market Performance.

	Insurers’ financial performance measures							
	Markup ($ per member month)		Premiums ($ per member month)		Claims ($ per member month)		ln(member-years), entire segment	
	Compliant	Noncompliant	Compliant	Noncompliant	Compliant	Noncompliant	Compliant	Noncompliant
State policy decisions								
Allow grandmothered plans	−12.1	34.6*	−7.3	−37.1**	27.0*	−69.1***	−0.15	1.42
	(6.5)	(16.2)	(7.0)	(10.9)	(11.5)	(12.7)	(0.10)	(0.96)
Expand Medicaid	9.0	23.5	2.6	34.9*	−25.3	−2.0	−0.03	−0.66
	(13.1)	(54.3)	(15.0)	(20.5)	(27.4)	(66.5)	(0.12)	(0.48)
State-based Marketplace	5.1	−16.7	17.4*	−10.1	17.4	9.7	−0.05	−0.14
	(8.4)	(75.4)	(8.7)	(11.1)	(30.5)	(81.9)	(0.13)	(0.22)
Insurer type								
Blue cross affiliated	26.4**	77.3***	23.8***	24.5	8.1	−52	NA	NA
	(6.5)	(16.6)	(6.1)	(24.1)	(19.5)	(35.3)		
Medicaid Managed Care	13.3*	51.3**	−7	−33.2	−30	−96.1*	NA	NA
	(5.3)	(17.9)	(9.4)	(30.7)	(34.2)	(38.3)		
Big Four	2.6	18.1	−15.4	−14.1	−47.0*	−21.8	NA	NA
	(8.1)	(13.4)	(10.3)	(35.4)	(27.2)	(45.9)		
Year								
2015	−12.9*	28.9**	12.3***	17.4*	18.1*	−10.5	0.56***	−0.53***
	(5.7)	(8.7)	(2.9)	(6.8)	(6.9)	(12.0)	(0.05)	(0.08)
2016	−13	54.4***	42.4***	52.4***	27.1*	−3.1	0.54***	−0.90***
	(9.5)	(15.1)	(5.6)	(9.5)	(12.9)	(13.8)	(0.10)	(0.12)
Observations	366	418	366	418	366	418	153	153

*Note.* Table reports the coefficients on the indicated variables, obtained from a regression of the indicated outcomes on those variables, for the indicated segment. Markups, premiums, and claims costs are per member month and measured at the insurer-segment level. Member-years are aggregated to the segment state. Also included, but not shown, are a full set of state fixed effects. The insurer financial performance sample is limited insurer-year-segments with at least 1000 member months, and weighted by enrollment. The reference categories for insurer type and year are all other insurers and 2014, respectively. Robust standard errors, clustered on state, are in parentheses.

* *P* < .05. **P < .01. ***P < .001.

Our first finding is that allowing grandmothered plans to continue to be offered was associated with a $35 increase in markups per member per month for noncompliant plans, and a $12 decrease for compliant plans. This comes from both a reduction in noncompliant plans’ claims costs and an increase in compliant plans’ costs. Allowing grandmothered plans in that state was associated with a $27 increase in compliant plans’ claims costs, and a $69 decrease in noncompliant plans’ claims costs per member month. This is consistent with prior research finding that allowing grandmothered plans was associated with higher costs relative to premiums for compliant plans.^6^ Overall, we find that the gap between compliant and noncompliant plans is strongly associated with state grandmothering decisions. This suggests that the presence of noncompliant ACA plans exacerbated the degree of selection in the risk pools of the compliant plans. This hypothesis implies that allowing grandmothered plans should have had a substantial impact on enrollment in the compliant and noncompliant segments. Looking at the natural log of enrollment, by segment, we find statistically insignificant but fairly large effects, with grandmothering reducing compliant enrollment by about 15% and having a very large (but noisy) impact on noncompliant enrollment.

State grandmothering decisions have an important association with individual market outcomes. Our results are more nuanced for other state policy decisions. Although we hypothesize that Medicaid expansions should reduce compliant plan premiums and costs, because low income is associated with poor health (so Medicaid expansion would pull expensive Marketplace enrollees into Medicaid),^23,24^ we find little evidence to support this hypothesis. Medicaid expansions were not statistically significantly associated with either compliant plan premiums or costs, although we find that Medicaid expansion was associated with a $35 increase in noncompliant plan premiums and a 5300 member-year decrease in enrollment in the noncompliant segment. The decrease in enrollment may have contributed to the increase in premiums in the noncompliant segment, if relatively healthier people dropped noncompliant coverage for Medicaid coverage. It may be surprising that Medicaid expansion could affect the noncompliant segment, but this finding suggests that at least some relatively low-income people retained noncompliant coverage, only to drop it upon attaining Medicaid eligibility. Having a state-based Marketplace was associated with a $17 increase in premiums in the compliant segment; these additional premium costs were not born by subsidized Marketplace enrollees because their subsidies rise with premiums. Overall, these other state-based policies were not strongly associated with the compliant or noncompliant segments.

Last, we find evidence of strong insurer type effects on financial performance. Blue Cross–affiliated insurers experienced higher markups ($26 and $77 in the compliant and noncompliant segments); higher compliant plan premiums ($24); and higher member-years of enrollment. Insurers that operate Medicaid managed care plans experienced higher compliant plan markups ($14 and $51) and lower noncompliant plan claims costs ($96), relative to other non-Blue Cross–affiliated insurers. These findings regarding insurer type, which are consistent with previous studies,^25,26^ may reflect the different structures and strategic goals of these types of insurers.

We investigated the sensitivity of our findings to specification and sample choices. We estimated unweighted models, which are shown in Online Appendix Table 1. Generally, the results are similar, although some of the coefficients changed because the unweighted results are more sensitive to extreme costs and premiums of small insurers. We also explored how the estimated effect of grandmothering varies as we drop each of the states that changed its grandmothering policy. The results, shown in Online Appendix Table 2, are fairly consistent, indicating that no single state drives our findings.

## Discussion

MLR filings show that noncompliant plans have low premiums, very low claims costs, and very high markups relative to compliant plans in the individual market. State policies, particularly decisions to allow grandmothered plans to operate, have a substantial influence on the difference in markups. These differences are likely due to differences in both the benefit designs and enrollee attributes and behaviors. Noncompliant plans are likely much less generous, as they need not provide essential health benefits nor offer the same level of financial protection as ACA-compliant plans. We caution, however, that an important limitation of our results is that only 3 states changed grandmothering regulations during our sample period (Colorado, New Mexico, and Oregon), so our findings are potentially sensitive to other changes in these states. In particular, Colorado and Oregon both saw their CO-OP health plans go bankrupt. Colorado’s sole CO-OP exited the market for 2016, the same year it disallowed grandmothered plans. Oregon, which also disallowed grandmothered plans in 2016, had 2 CO-OPs; one went bankrupt in late 2014 and the other in mid-2016. If anything, these bankruptcies would increase compliant-segment premiums (as CO-OPs tended to be low premium), making it harder for us to detect effects of grandmothering. Another limitation is the generalizability of results from our time period, as the individual mandate has been repealed and many insurers have exited. However, the insight our evidence provides on performance of noncompliant plans and their negative effects on compliant plans is vital to keep in mind as regulators adjust to new circumstances.

Noncompliant plans are likely attractive for individuals in relatively good health, because these plans offer low premiums in exchange for low benefits. Indeed, evidence comparing Marketplace and off-Marketplace enrollees—a comparison which is similar to but not identical to the comparison of compliant and noncompliant plans—shows that off-Marketplace enrollees are healthier on many dimensions. They are more likely to report being in excellent health, and they are less likely to smoke, be obese, or have hypertension or diabetes.^27^ This favorable selection also reflects the fact that off-Marketplace and noncompliant plans are ineligible for premium tax credit subsidies, making them especially unappealing to lower income households who may have greater health needs.^23,24^ A final factor suggesting that noncompliant plans are disproportionately likely to enroll healthier people is the fact that many of these plans predate the ACA regulations of guaranteed issue and modified community rating. Enrollees in these plans could therefore have been subject to medical underwriting in most states to ensure they did not have preexisting conditions.

We conclude that the low claims costs and high markups of noncompliant plans are likely due to cream-skimming, meaning they disproportionately enroll healthy, low-cost people in the 2010-2013 period when they were taking new enrollees, and retained healthy enrollees throughout the 2014-2016 period. The economics of cream-skimming imply that less generous plans offer lower premiums and attract healthier enrollees, driving up the premiums of more generous plans, and ultimately reducing overall coverage and enrollment in the most comprehensive plans.^28^

Although this cream-skimming may result from grandfathered and grandmothered plans with declining enrollment, it may have important implications given recent policy changes. Specifically, beginning in 2018, the Trump Administration has allowed the sale of short-term insurance plans and association health plans. Short-term plans are currently expanding; association health plans are currently under legal challenge.^29^ Neither of these types of plans is subject to the ACA’s guaranteed issue or underwriting regulations, nor must they offer minimum essential coverage. Furthermore, as noncompliant, nongrandfathered, or grandmothered plans do not count as coverage for the individual mandate, noncompliant plans may also become more attractive with the individual mandate penalty set to zero for 2019 and beyond. Some new enrollment in these plans may well come from people who would otherwise be uninsured. It is also possible, however, that new growth in the noncompliant segment could occur at the expense of the compliant segment, particularly among high-income individuals who do not qualify for advance premium tax credits. If so, we would expect the presence of these plans to result in higher costs and premiums for Marketplace plans. Of course, these new noncompliant plans are different from grandmothered plans in several ways, and our results may not generalize to them. Importantly, grandmothered plans, by definition, already had enrollees prior to the ACA, and carried this enrollment forward. This may have made it easier for them to attract enrollees who might have otherwise signed up for compliant plans. On the contrary, enrollment in grandmothered plans was limited to previously enrolled individuals, whereas anyone will be able to sign up for short-term and association health plans if they qualify.

The potentially higher premiums generated by noncompliant plans have several important consequences. First, unsubsidized enrollees will face higher premiums for ACA-compliant coverage. As this coverage is more generous than noncompliant coverage, the ultimate consequences may be reductions in the quantity or quality of coverage. Incremental efforts to increase affordability through noncompliant plans may reduce affordability for unsubsidized enrollees with preexisting medical conditions, in particular. Second, for subsidized enrollees, the size of the subsidies rise one-for-one with the benchmark premium, so the out-of-pocket premium of the benchmark plan will not rise, but relative premiums of other plans may change. Thus, subsidized enrollees may substitute toward less generous plans.^30^ Finally, taxpayers ultimately pay for these subsidies and so a third consequence of higher compliant plan premiums is greater government spending.

## Supplemental Material

Appendix – Supplemental material for Same Game, Different Names: Cream-Skimming in the Post-ACA Individual Health Insurance Market Click here for additional data file. Supplemental material, Appendix for Same Game, Different Names: Cream-Skimming in the Post-ACA Individual Health Insurance Market by Daniel W. Sacks, Coleman Drake, Jean M. Abraham and Kosali Simon in INQUIRY: The Journal of Health Care Organization, Provision, and Financing

supplemental_material_2 – Supplemental material for Same Game, Different Names: Cream-Skimming in the Post-ACA Individual Health Insurance Market Click here for additional data file. Supplemental material, supplemental_material_2 for Same Game, Different Names: Cream-Skimming in the Post-ACA Individual Health Insurance Market by Daniel W. Sacks, Coleman Drake, Jean M. Abraham and Kosali Simon in INQUIRY: The Journal of Health Care Organization, Provision, and Financing
