# Supplementary material for: Same Game, Different Names: Cream-Skimming in the Post-ACA Individual Health Insurance Market
Source: Inquiry. 2020 Jul 9;57:0046958020933765. doi: 10.1177/0046958020933765 (PMC7357013; doi:10.1177/0046958020933765)
Supplement: Appendix – Supplemental material for Same Game, Different Names: Cream-Skimming in the Post-ACA Individual Health Insurance Market [file Appendix.pdf]

Appendix Figure 1. Median medical loss ratios by individual market segment

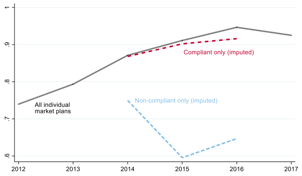

Note. Figure report the median medical loss ratio (across insurers within a given year and segment). For all individual market plans, the medical loss ratio is directly observed. For segment-specific ratios, we impute the loss ratio as follows. The numerator is claims costs less ACA-related transfers received (net payments for reinsurance, risk adjustment, risk corridors, and cost-sharing reductions). The denominator is premiums earned less taxes. We do not observe segment specific taxes so we allocate overall individual market taxes according to each segment's enrollment share. Compliant and non-compliant statistics are only reported for 2014-2016 because those are the years in which Risk Corridor payment data were available.

Appendix Table 1. Associations between State Policy Decisions and Individual Market Insurers' Financial Performance, Unweighted

	Financial Performance Measure, Compliant non-Compliant plans							
	Markup ($ per member month)		Premiums ($ per member month)		Claims ($ per member month)		Member-Years (1,000s)	
	Compliant	Non	Compliant	Non	Compliant	Non	Compliant	Non
State Policy Decisions								
Allow Grandmothered	-13.7 **	-11.7	-20.2	-0.2	27.9 *	10.5	1.6	3.5
Plans	(6.5)	(23.4)	(13.3)	(27.7)	(14.1)	(41.2)	(5.4)	(2.7)
Expand Medicaid	13.1	34.7	57.4	42.6 **	33.4	-6.4	3.6	-5.3 **
	(23.1)	(92.4)	(37.9)	(18.9)	(24.1)	(103.4)	(5.8)	(2.1)
State-Based	-12.7	92.4	-16.1	-28.2 **	-21.6	-111.5	-1.2	0.2
Marketplace	(13.3)	(101.4)	(25.5)	(11.4)	(25.3)	(106.0)	(4.0)	(2.0)
Insurer Type								
Blue Cross Affiliated	-2.1	104.5 **	5.2	19.0 **	3.2	-87.3 *	12.6 ***	-2.9 ***
	(6.1)	(42.1)	(3.6)	(7.9)	(8.7)	(43.7)	(2.0)	(0.9)
Medicaid Managed	-4	73.0 ***	26.9 ***	42.2 ***	-0.6	-35.7	13.6 ***	-4.5 ***
Care	(7.8)	(23.0)	(6.0)	(10.5)	(11.5)	(25.6)	(3.1)	(1.6)
Big Four	-2.1	104.5 **	5.2	19.0 **	3.2	-87.3 *	12.6 ***	-2.9 ***
	(6.1)	(42.1)	(3.6)	(7.9)	(8.7)	(43.7)	(2.0)	(0.9)
Year								
2015	-2.1	104.5 **	5.2	19.0 **	3.2	-87.3 *	12.6 ***	-2.9 ***
	(6.1)	(42.1)	(3.6)	(7.9)	(8.7)	(43.7)	(2.0)	(0.9)
2016	-4	73.0 ***	26.9 ***	42.2 ***	-0.6	-35.7	13.6 ***	-4.5 ***
	(7.8)	(23.0)	(6.0)	(10.5)	(11.5)	(25.6)	(3.1)	(1.6)
Insurer-Years	366	418	366	418	366	418	366	418

Note. Table reports the coefficients on the indicated variables, obtained from a regression of the indicated outcomes on those variables, for the indicated segment. Also included, but not shown, are a full set of state fixed effects. The reference categories for insurer type and year are all other insurers and 2014, respectively. The sample is limited to insurer-year-segments with at least 1,000 member-years. Robust standard errors, clustered on state, are in parentheses. * $p < 0.05$ ** $p < 0.01$ *** $p < 0.001$

Appendix Table 2. Sensitivity of Grandmothering Coefficient to Inclusion of Individual States

	Insurers' Financial Performance Measures						ln(Member-Years), entire segment	
	Markup ($ per member month)		Premiums ($ per member month)		Costs ($ per member month)			
Sample	Compliant	Non	Compliant	Non	Compliant	Non	Compliant	Non
All states	-12.1*	34.6**	-7.3	-37.1***	27.0**	-69.1***	-0.15	1.42
	(6.5)	(16.2)	(7.0)	(10.9)	(11.5)	(12.7)	(0.10)	(0.96)
Drop CO	-13.5*	48.6***	-8.6	-21.7***	19.0*	-68.0***	-0.14	1.77
	(6.8)	(14.4)	(10.3)	(7.6)	(10.7)	(13.1)	(0.12)	(1.32)
Drop NM	-12.8*	34.6**	-10.8*	-37.1***	26.1**	-69.1***	-0.11	0.49**
	(6.5)	(16.2)	(6.2)	(10.9)	(11.8)	(12.1)	(0.11)	(0.20)
Drop OR	-9.8	27.2*	-0.3	-45.4***	35.9***	-69.8***	-0.22***	1.98*
	(6.4)	(14.6)	(7.9)	(7.9)	(9.4)	(12.6)	(0.08)	(1.14)

Notes. Table reports the coefficient on a dummy variable for "allows Grandmothered plans," obtained from a regression of the indicated outcome (in the indicated segment) on the Grandmothering indicator, as well as indicators for Medicaid expansion, state based marketplace, insurer type, as well as year and state fixed effects. Markups, premiums, and claims costs are per member month and measured at the insurer-segment level. Member-years are aggregated to the segment-state. The insurer financial performance sample is limited insurer-year-segments with at least 1000 member months, and weighted by enrollment. The reference categories for insurer type and year are all other insurers and 2014, respectively. Robust standard errors, clustered on state, are in parentheses.* $p < 0.1$ ** $p < 0.05$ *** $p < 0.001$
