# Supplementary material for: Same Game, Different Names: Cream-Skimming in the Post-ACA Individual Health Insurance Market
Source: Inquiry. 2020 Jul 9;57:0046958020933765. doi: 10.1177/0046958020933765 (PMC7357013; doi:10.1177/0046958020933765)
Supplement: supplemental_material_2 – Supplemental material for Same Game, Different Names: Cream-Skimming in the Post-ACA Individual Health Insurance Market [file supplemental_material_2.pdf]

Additional details on state grandmothering decisions

Information on state grandmothering decisions were obtained from the website healthinsurance.org. The website indicates, for each state, whether grandmothered plans are allowed, as of 2019. We code state grandmothering decisions each year by whether they allowed grandmothered plans for the entirety of that year. We confirmed the supporting documentation from healthinsurance.org for the 40 states for which it is available. This documentation is typically a bulletin, memorandum, or press release from the state insurance regulator, indicating the state's decision to allow or disallow non-compliant plans. In 11 cases no explicit documentation is available. For five states that allowed grandmothered plans throughout our sample period (Georgia, Kansas, Pennsylvania, Montana, and Tennessee), healthinsurance.org reports that the states have confirmed the reported policy. Documentation is also unavailable for six states that did not permit renewal of grandmothered plans by the end of our sample period: New York, Oregon, Rhode Island, Washington, and Vermont.

Because supporting documentation is not always available, we also used enrollment data to help validate our grandmothering measure. Specifically, if we are measuring grandmothering appropriately, then the share of covered lives in non-compliant plans should be higher in states that allow grandmothered plans than in states that do not. Appendix Figure 1 plots this share over time for five groups of states: States that always allowed grandmothering (with verification), states that always allowed grandmothering (without verification), states that never allowed grandmothering (with verification), and states that switched, disallowing grandmothered plans after 2014 or after 2015. The figure provides reassuring evidence on the validity of our measure. Non-compliant plan enrollment is much higher in the states that allow grandmothered plans than in states that do not. Unverified states look like verified states. Switcher states start off with high rates of non-compliant plan enrollment, but end up with low rates once they have switched.

Supplemental Figure 1. Non-compliant plan share of insurance coverage

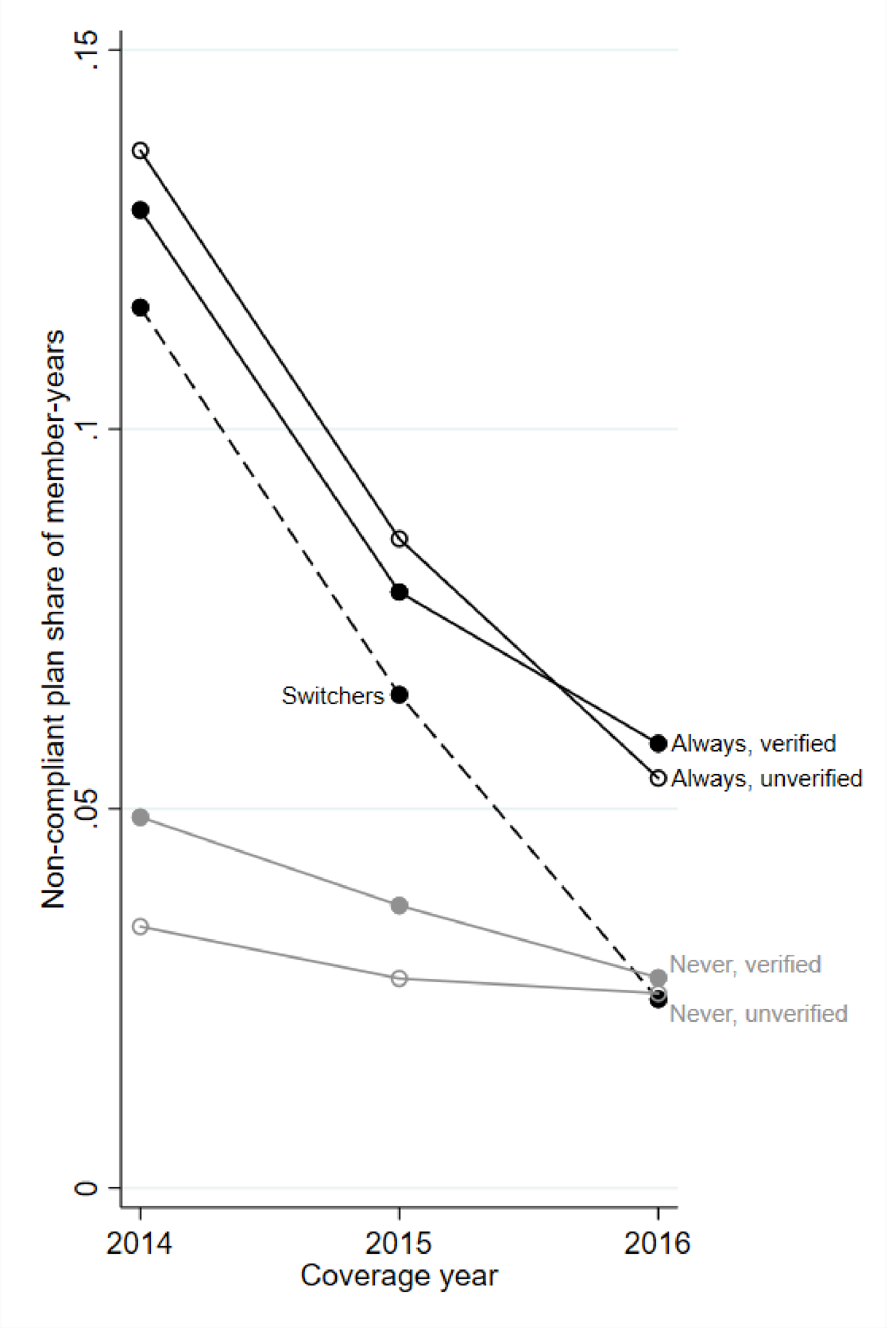

Notes: Figure shows, for each group and year, the share of insurance coverage in non-compliant plans, among plans in the MLR filing data. “Always” refers to states which always allowed grandmothered plans over the 2014-2016 period. “Never” refers to states which never allowed grandmothered plans, and “switchers” refers to states which switched, allowing grandmothered plans in 2014 but not by 2016. “Verified” are states with explicit documentation of their grandmothering rules; “unverified” are states without such documentation.
